# Supplementary material for: The Steric Effect in Preparations of Vanadium(II)/(III) Dinitrogen Complexes of Triamidoamine Ligands Bearing Bulky Substituents
Source: Molecules. 2022 Sep 9;27(18):5864. doi: 10.3390/molecules27185864 (PMC9500765; doi:10.3390/molecules27185864)
Supplement: Supplementary file 1 [file molecules-27-05864-s001.zip › Molecules_SI.pdf]

Supplementary Materials

The Steric Effect in Preparations of Vanadium(II)/(III) Dinitrogen Complexes of Triamidoamine Ligands Bearing Bulky Substituents

Contents:

**Table S1.**	Experimental Data for X-ray Diffraction Studies on Crystalline Complexes **1**, **2**, **3**, and **4**	S3
**Figure S1**.	Raman spectra of **1** prepared under $^{14}N_2$ and $^{15}N_2$ in toluene (λ_{ex} = 532 nm) at room temperature and their difference spectrum.	S4
**Figure S2**.	Raman spectra of **2** prepared under $^{14}N_2$ and $^{15}N_2$ in toluene (λ_{ex} = 532 nm) at room temperature and their difference spectrum.	S5
**Figure S3.**	IR spectra (ATR) of **1** prepared under $^{14}N_2$ and $^{15}N_2$.	S6
**Figure S4.**	IR spectra (ATR) of **2** prepared under $^{14}N_2$ and $^{15}N_2$.	S6
**Figure S5.**	IR spectra (ATR) of **4** prepared under $^{14}N_2$ (black line) and $^{15}N_2$ (red line).	S7
**Figure S6**.	^{1}H NMR spectrum of [{V($\mathbf{L}^{iPr}$)}$_2$(μ-N_2)] (**1**) in C_6D_6 at 298 K.	S8
**Figure S7**.	^{1}H NMR spectrum of [{V($\mathbf{L}^{Pen}$)}$_2$(μ-N_2)] (**2**) in C_6D_6 at 298 K.	S8
**Figure S8.**	^{15}N NMR spectrum of [{V$\mathbf{L}^{iPr}$}$_2$(μ-$^{15}N_2$)] (**1′**) in C_6D_6 at 298 K.	S9
**Figure S9.**	^{15}N NMR spectrum of [{V$\mathbf{L}^{pen}$}$_2$(μ-$^{15}N_2$)] (**2′**) in C_6D_6 at 298 K.	S9
**Figure S10**.	^{51}V NMR spectrum of [{V($\mathbf{L}^{iPr}$)}$_2$(μ-N_2)] (**1**) in C_6D_6 at 298 K.	S10
**Figure S11**.	^{51}V NMR spectrum of [{V($\mathbf{L}^{pen}$)}$_2$(μ-N_2)] (**2**) in C_6D_6 at 298 K.	S10
**Figure S12**.	^{1}H NMR spectrum of [V($\mathbf{L}^{Cy2}$)] (**3**) in C_6D_6 at 298 K.	S11
**Figure S13**.	^{1}H NMR spectrum of [VK($\mathbf{L}^{Cy2}$)(μ-N_2)(18-crown-6)] (**4**) in C_6D_6 at 298 K.	S12
**Figure S14**.	^{1}H NMR spectra of $H_3\mathbf{L}^{Cy2}$ and [VK($\mathbf{L}^{Cy2}$)(μ-N_2)(18-crown-6)] (**4**) in the range of –2 – 10 ppm in C_6D_6 at 298 K.	S12
**Figure S15**.	^{1}H NMR spectrum of $^{14}NH_4^+$ that was obtained from the reaction of **1** with 80 equiv. K[$C_{10}H_8$] and 80 equiv. HOTf under $^{14}N_2$.	S13
**Table S2.**	Yields of NH_3 and N_2H_4 Produced by the Protonation of Dinitrogen Ligands for **1**, **2**, **3**, and **4**.	S14
**Figure S16**.	Calibration curves for hydrazine quantification.	S15

Table S1. Experimental Data for X-ray Diffraction Studies on Crystalline Complexes **1**, **2**, **3**, and **4**

compound	**1**	**2**	**3**	**4**
formula	$C_{30}H_{66}N_{10}V_2$	$C_{42}H_{90}N_{10}V_2$	$C_{45}H_{81}N_4V$	$C_{68}H_{128}KN_6O_8V$
formula weight	668.80	845.86	729.07	1219.87
crystal system	Orthorhombic	Monoclinic	Monoclinic	Monoclinic
space group	*Cmca*	*C*2/*c*	*P*2$_1$/*c*	*P*2$_1$/*c*
a [Å]	15.9064(4)	16.8740(6)	10.8057(2)	13.9446(3)
b [Å]	12.2273(4)	19.0029(7)	20.5056(4)	22.9058(4)
c [Å]	19.1253(5)	14.4824(5)	19.1296(4)	22.4762(4)
α[˚]	90	90	90	90
β[˚]	90	91.324(6)	97.359(7)	99.918(7)
γ[˚]	90	90	90	90
V [Å^3]	3719.72(18)	4642.6(3)	4203.77(16)	7071.9(3)
Z	4	4	4	4
temp [K]	173	173	173	173
λ[Å]	0.71073	0.71073	0.71073	1.54178
ρ_{calc} [g cm^{-3}]	1.194	1.210	1.152	1.172
μ [mm^{-1}]	0.536	0.444	0.271	2.215
No. of reflections	17179	22176	39808	12841
No. of independent reflections [R(int)]	2191 (0.051)	5308 (0.071)	9593 (0.042)	12841 (0.047)
No. of parameters	121	250	451	846
R_1 ($I > 2\sigma(I)$)	0.0408	0.0442	0.0435	0.0616
wR_2 (all data)	0.0878	0.0972	0.043	0.1651
goodness-of-fit on F^2	1.083	1.030	1.035	1.031
largest diff. peak/hole [eÅ^{-3}]	0.38 / -0.22	0.54 / -0.22	0.32 / -0.38	0.565 / -0.561

[a] $R_1 = \Sigma||F_0| - |F_c|| / \Sigma|F_0|$ for $F_0 > 2\sigma(F_0)$. [b] $wR_2 = [\Sigma w(F_0^2 - F_c^2)^2 / \Sigma w(F_0^2)^2]^{1/2}$.

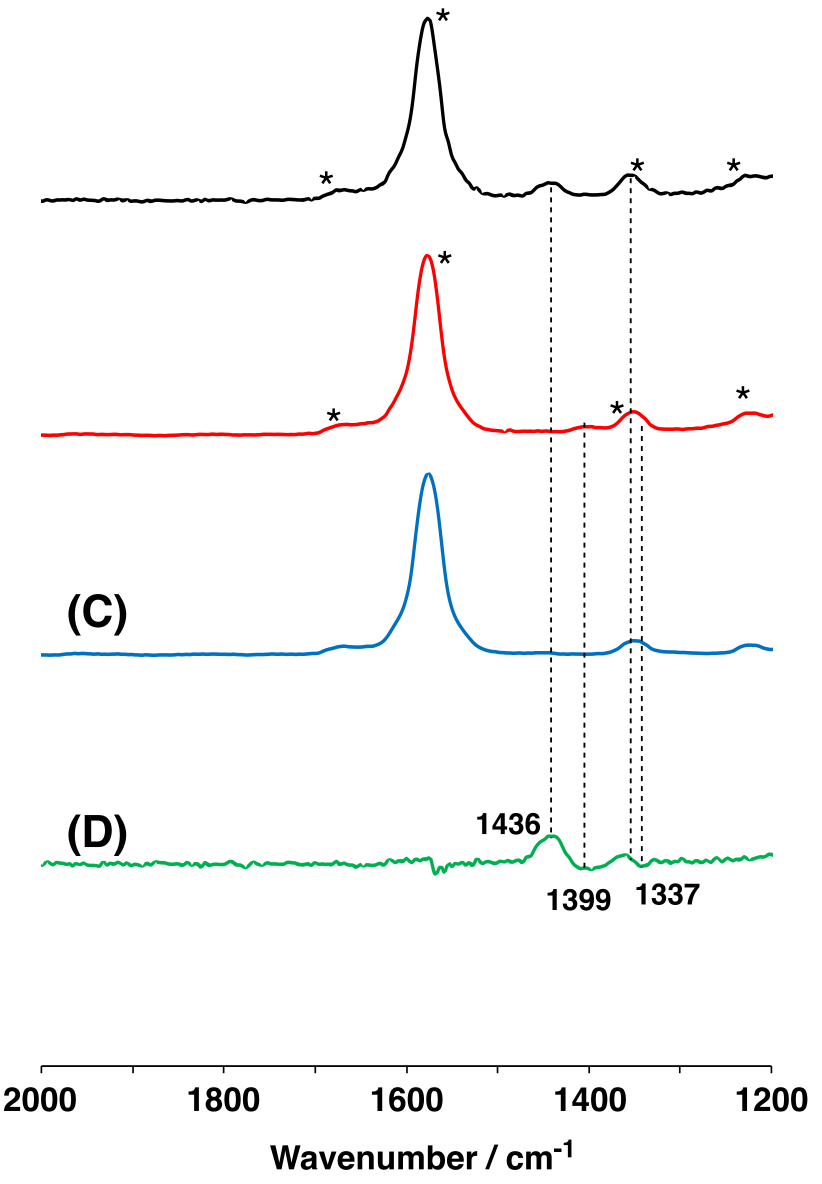

Figure S1. Raman spectra of **1** prepared under $^{14}N_2$ (spectrum A) and $^{15}N_2$ (spectrum B) in toluene as solvent (spectrum C) (λ_{ex} = 532 nm) at room temperature and their difference spectrum (spectrum D). Peaks with asterisks indicate those of toluene as a solvent.

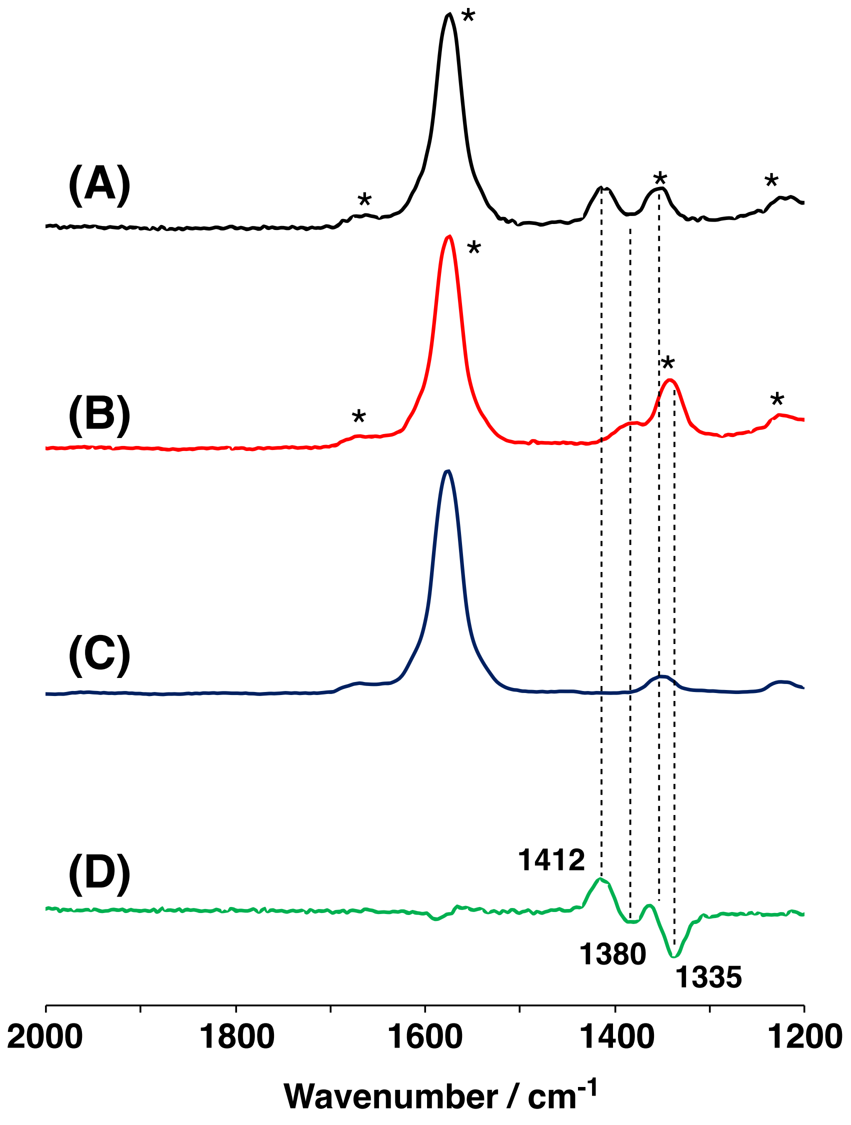

Figure S2. Raman spectra of **2** prepared under $^{14}N_2$ (spectrum A) and $^{15}N_2$ (spectrum B) in toluene as solvent (spectrum C) (λ_{ex} = 532 nm) at room temperature and their difference spectrum (spectrum D). Peaks with asterisks indicate those of toluene as a solvent.

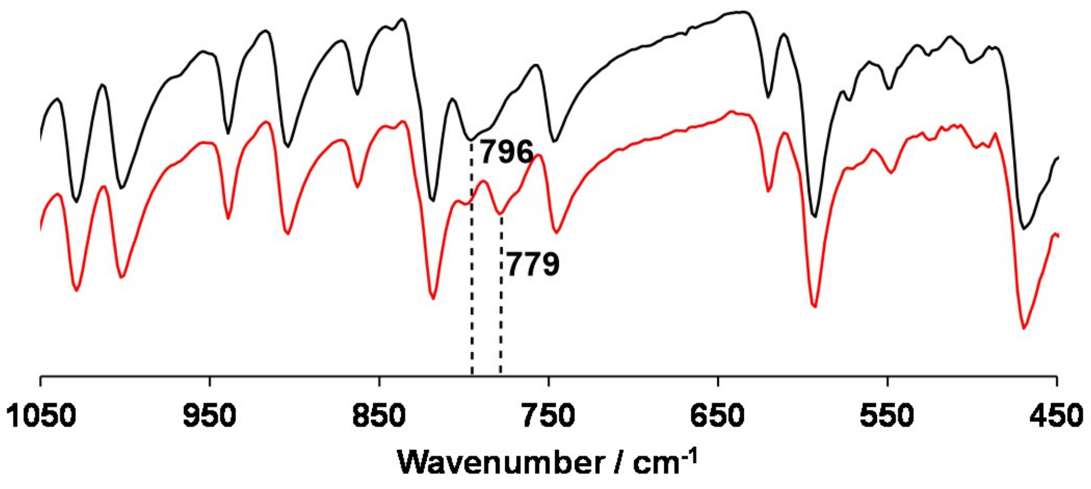

Figure S3. IR spectra (ATR) of **1** prepared under $^{14}N_2$ (black line) and $^{15}N_2$ (red line).

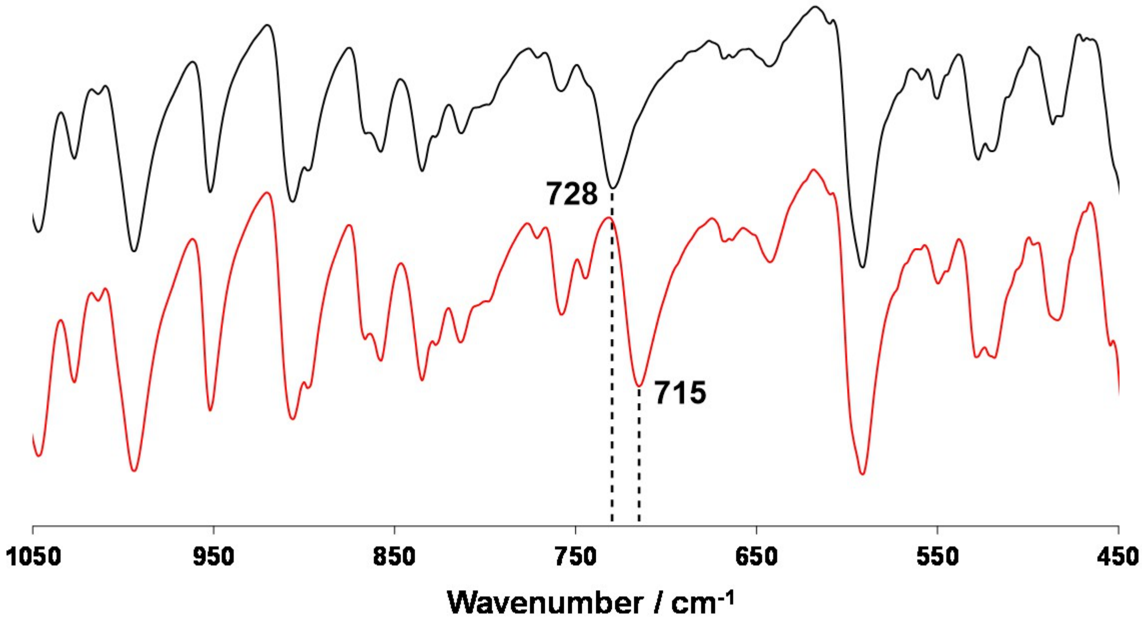

Figure S4. IR spectra (ATR) of **2** prepared under $^{14}N_2$ (black line) and $^{15}N_2$ (red line).

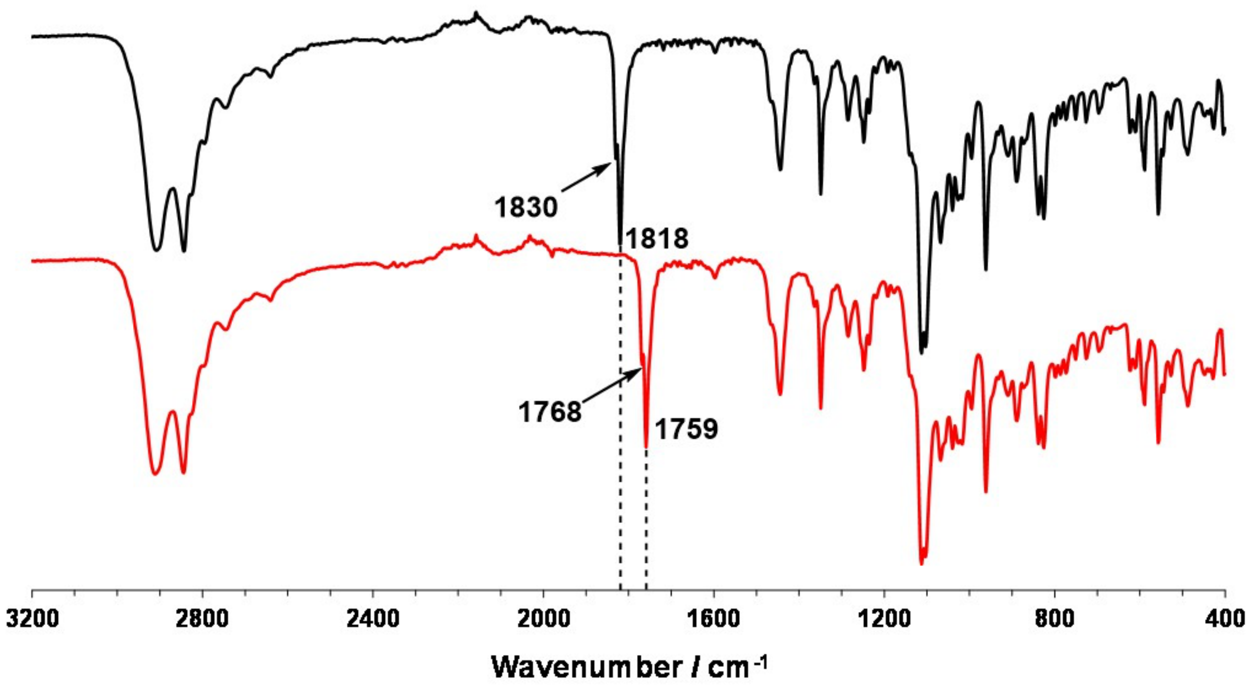

Figure S5. IR spectra (ATR) of **4** prepared under $^{14}N_2$ (black line) and $^{15}N_2$ (red line).

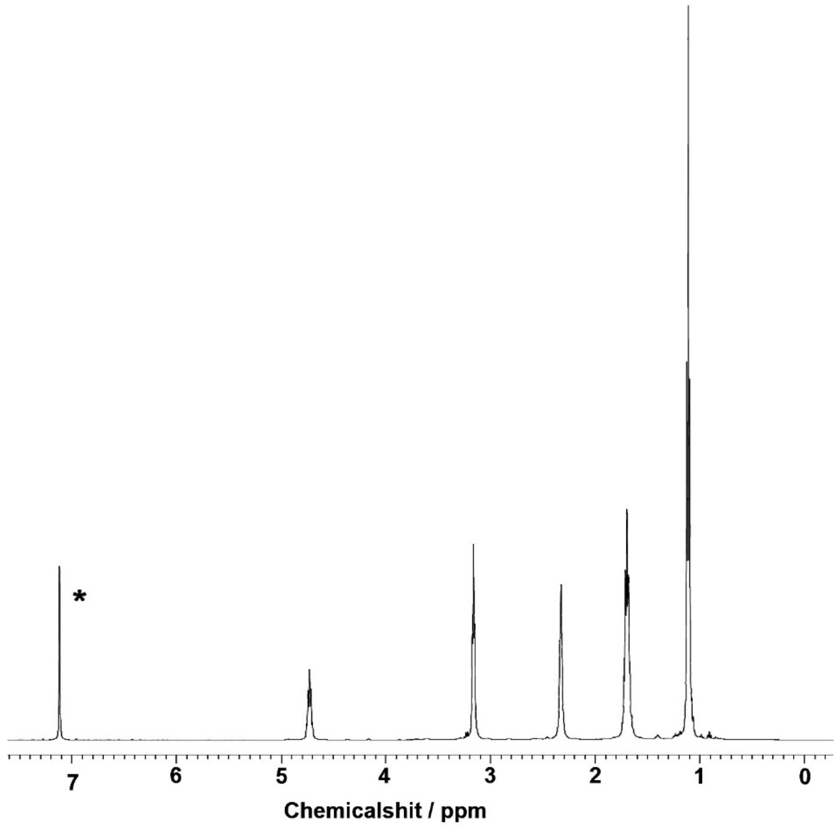

Figure S6. ^{1}H NMR spectrum of $[\{V(\mathbf{L^{iPr}})\}_2(\mu\text{-}N_2)]$ (**1**) in C_6D_6 at 298 K (500 MHz, δ/ppm *vs* C_6D_6 (7.16 ppm)). The peak with an asterisk shows solvent.

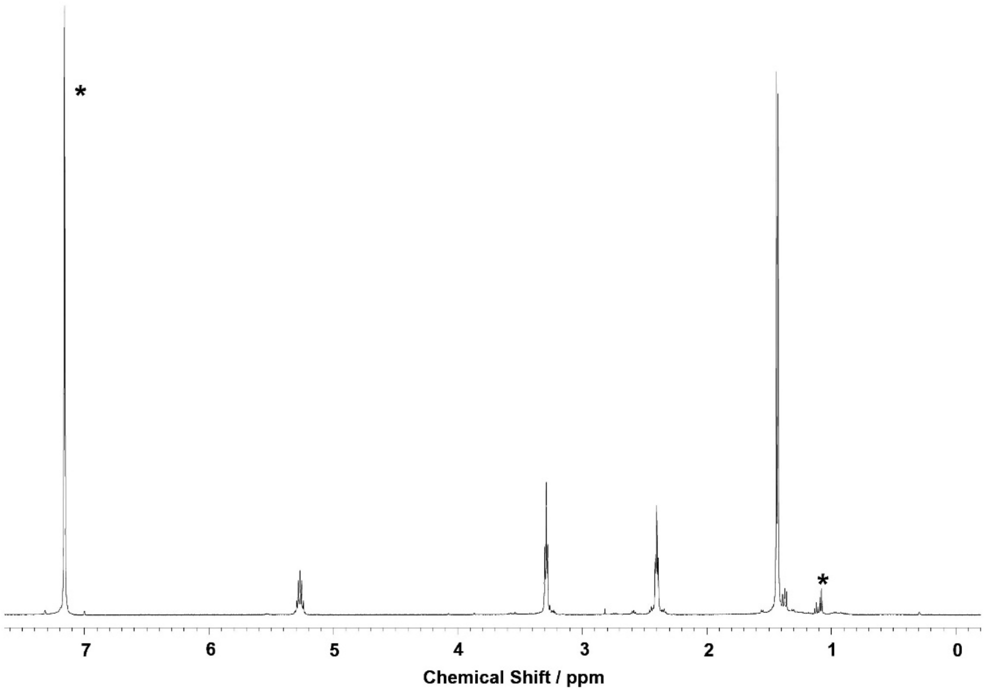

Figure S7. ^{1}H NMR spectrum of $[\{V(\mathbf{L^{Pen}})\}_2(\mu\text{-}N_2)]$ (**2**) in C_6D_6 at 298 K (500 MHz, δ/ppm *vs* C_6D_6 (7.16 ppm)). The peaks with an asterisk show those of solvent and small amounts of impurities.

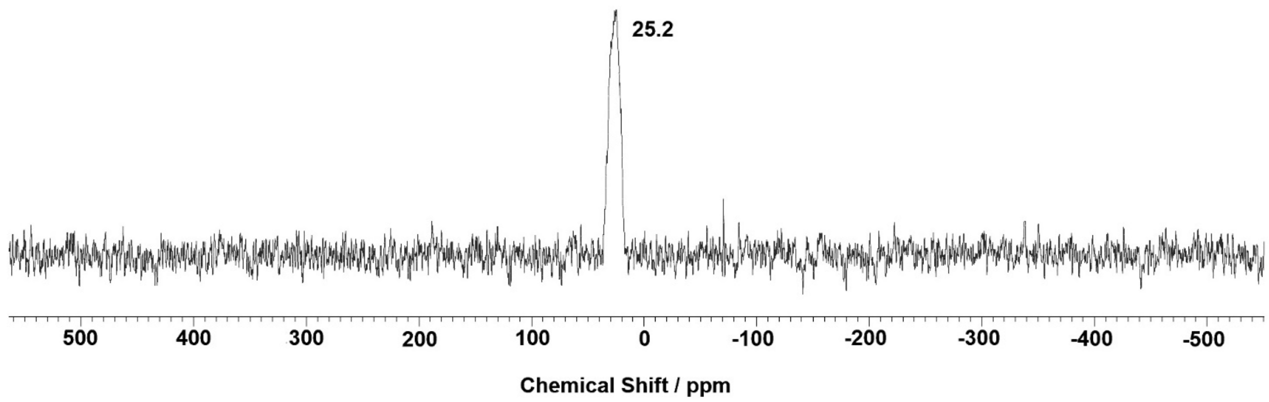

Figure S8. ^{15}N NMR spectrum of $[\{VL^{iPr}\}_2(\mu\text{-}^{15}N_2)]$ (**1′**) in C_6D_6 at 298 K (60.815 MHz, δ/ppm *vs* CH_3NO_2 (0.00 ppm)).

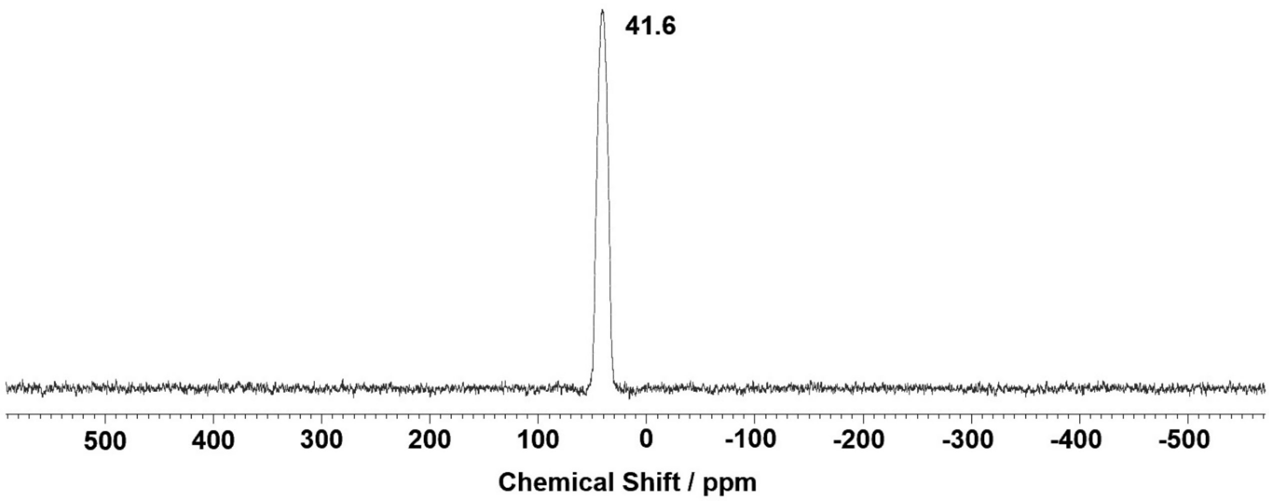

Figure S9. ^{15}N NMR spectrum of $[\{VL^{pen}\}_2(\mu\text{-}^{15}N_2)]$ (**2′**) in C_6D_6 at 298 K (60.815 MHz, δ/ppm *vs* CH_3NO_2 (0.00 ppm)).

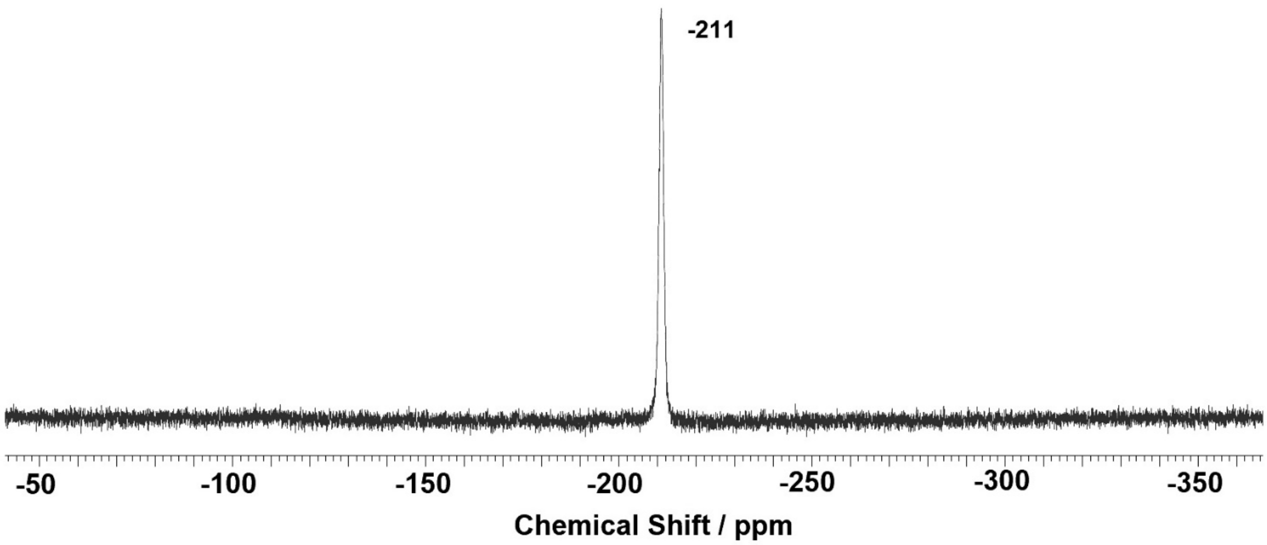

Figure S10. ^{51}V NMR spectrum of [{V($\mathbf{L}^{\mathbf{iPr}}$)}$_2$($\mu$-$N_2$)] (**1**) in C_6D_6 at 298 K (131.56 MHz, δ/ppm *vs* $VOCl_3$ (0.00 ppm)).

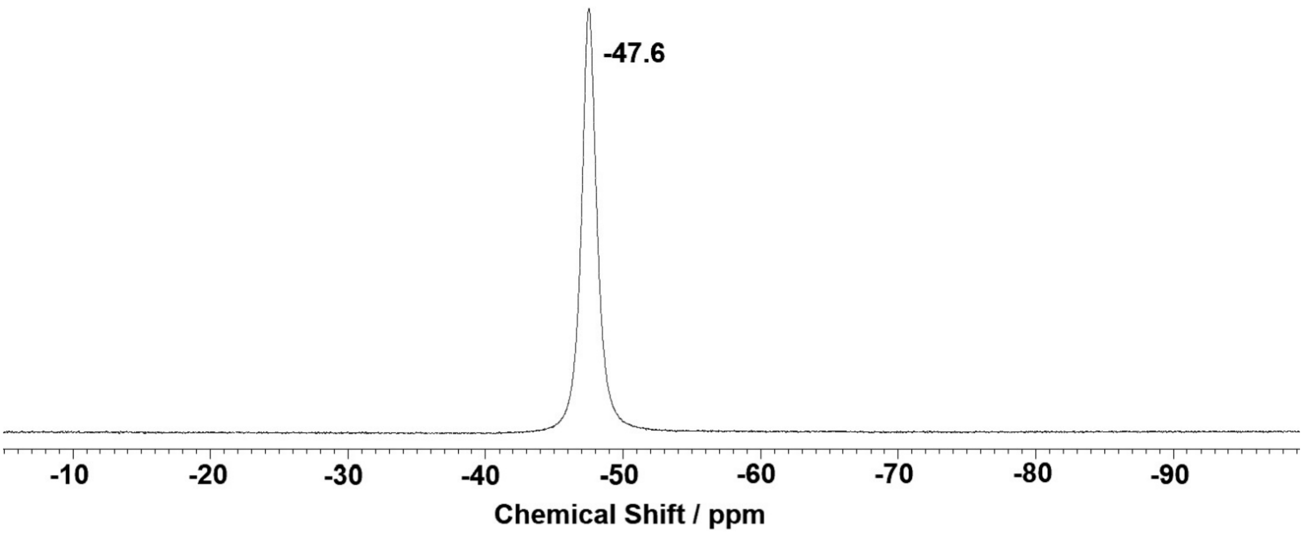

Figure S11. ^{51}V NMR spectrum of [{V($\mathbf{L}^{\mathbf{pen}}$)}$_2$($\mu$-$N_2$)] (**2**) in C_6D_6 at 298 K (131.56 MHz, δ/ppm *vs* $VOCl_3$ (0.00 ppm)).

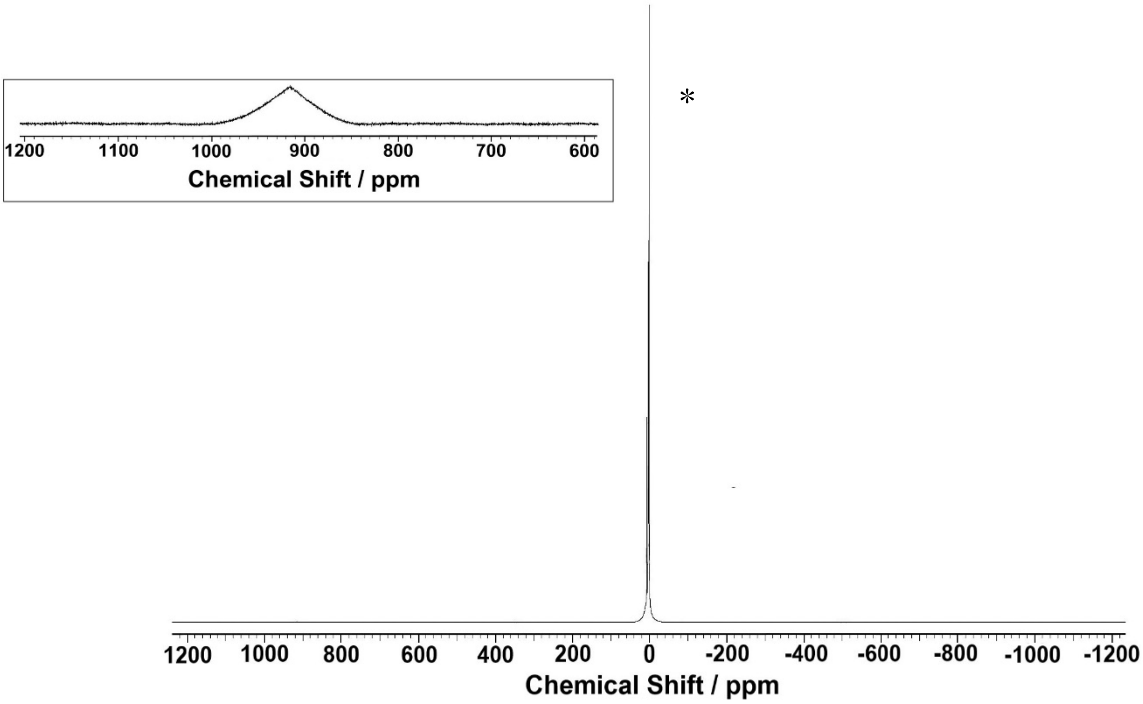

Figure S12. ^{1}H NMR spectrum of [V(**L**^{Cy2})] (**3**) in C_6D_6 at 298 K (500 MHz, *δ*/ppm *vs* C_6D_6 (7.16 ppm)). The peak with an asterisk shows solvent. Inset: Expanded views of a range of 1200 – 600 ppm (top).

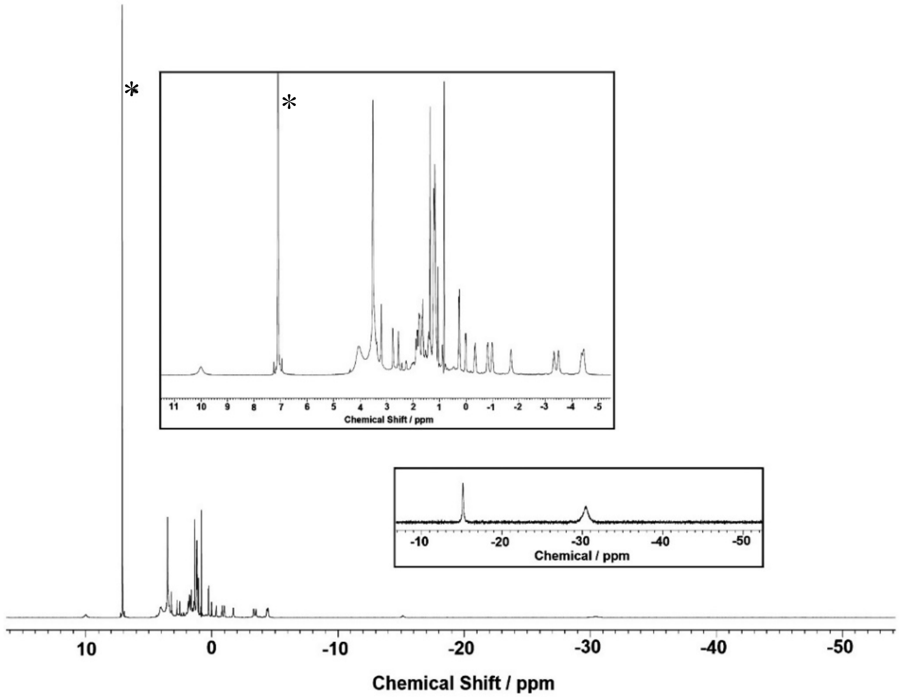

Figure S13. ^{1}H NMR spectrum of [VK(L^{Cy2})(μ-N_2)(18-crown-6)] (**4**) in C_6D_6 at 298 K (500 MHz, δ/ppm *vs* C_6D_6 (7.16 ppm)). The peak with an asterisk shows solvent. Inset: Expanded views of a range of –5 – 11 ppm (top) and –50 – –10 ppm (bottom).

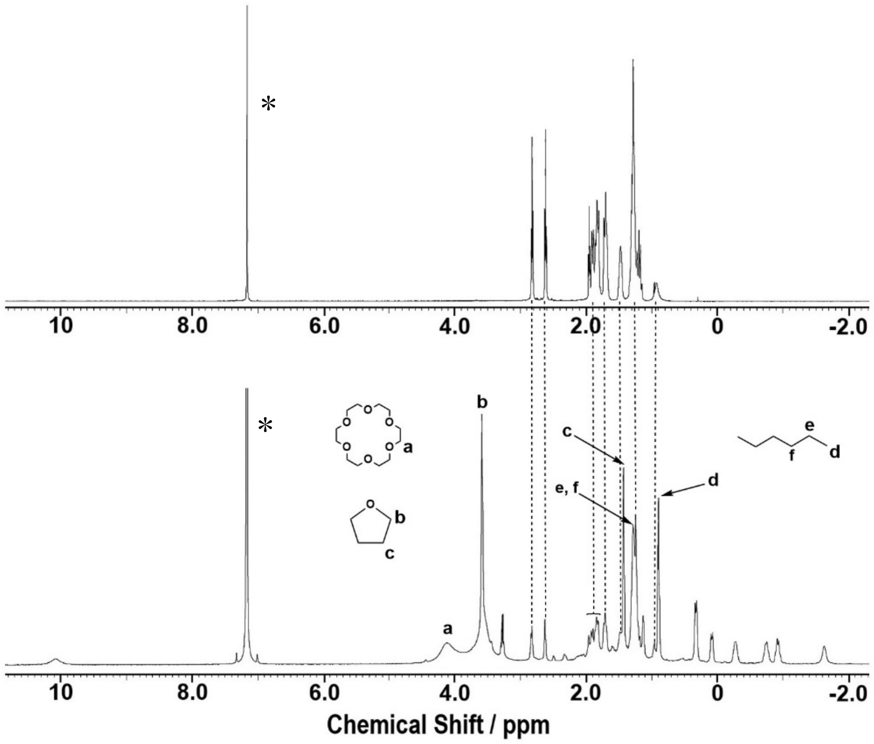

Figure S14. ^{1}H NMR spectra of H_3L^{Cy2} (top) and [VK(L^{Cy2})(μ-N_2)(18-crown-6)] (**4**) (bottom) in the range of –2 – 10 ppm in C_6D_6 at 298 K (500 MHz, δ/ppm *vs* C_6D_6 (7.16 ppm)). The peaks with an asterisk show solvent.

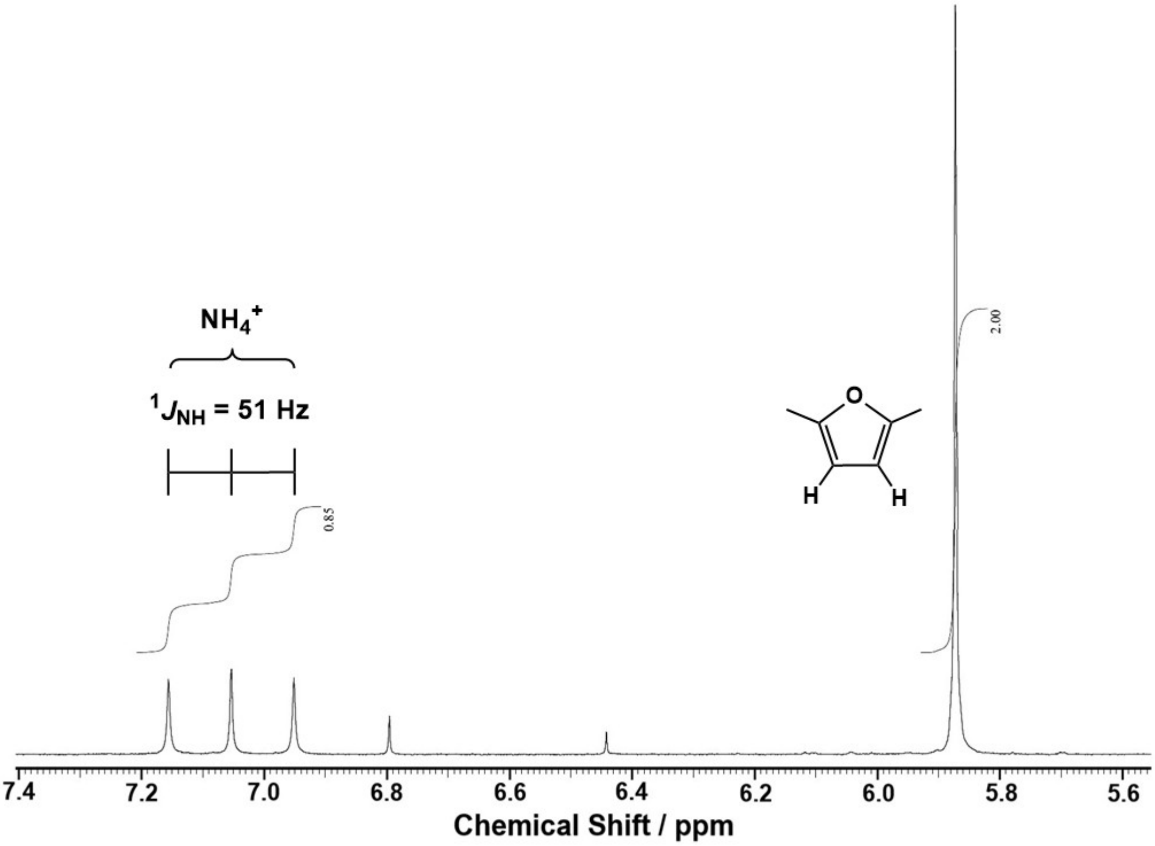

Figure S15. ^1H NMR spectrum of $^{14}NH_4^+$ that was obtained from the reaction of **1** with 80 equiv. K[$C_{10}H_8$] and 80 equiv. HOTf under $^{14}N_2$ (DMSO-d_6, 500 MHz). Chemical shifts are shown versus DMSO-d_6 (2.50 ppm).

Table S2. Yields of NH_3 and N_2H_4 Produced by the Protonation of Dinitrogen Ligands for **1**, **2**, **3**, and **4**.

complex[b]	reductant	proton source	product					
			NH_4^+[c]			N_2H_4[f]		
			Integration[d]	yield / %[e]	average	Absorbance[g]	yield / %[h] (calibration curve)	average
**1**	$Na^+[C_{10}H_8]^-$	HOTf	0.085	7.4	7.7	0.084	0.35 (1)	0.36
			0.082	7.1		0.084	0.35 (1)	
			0.10	8.7		0.096	0.40 (1)	
	$K^+[C_{10}H_8]^-$	HOTf	0.53	46.1	47.3	3.2	10.5 (2)	10.9
			0.50	43.5		3.3	11.1 (2)	
			0.82	52.2		3.4	11.2 (2)	
**2**	$Na^+[C_{10}H_8]^-$	HOTf	0.069	7.6	5.4	0.23	9.8 (1)	10.6
			0.040	4.4		0.25	10.7 (1)	
			0.038	4.2		0.26	11.2 (1)	
	$K^+[C_{10}H_8]^-$	HOTf	0.36	39.6	37.8	3.7	15.4 (2)	15.9
			0.51	44.0		3.7	15.6 (2)	
			0.27	29.7		3.9	16.7 (2)	
**3**	$Na^+[C_{10}H_8]$	HOTf	0.026	4.9	6.5	-0.0034	- 0.0003 (1)	n.d.
			0.026	5.0		-0.00099	- 0.0001 (1)	
			0.051	9.6		-0.00054	0.0000 (1)	
	$K^+[C_{10}H_8]^-$	HOTf	0.79	74.9	77.4	0.85	6.22 (2)	6.7
			0.81	76.8		0.94	6.83 (2)	
			0.85	80.6		0.95	6.92 (2)	
**4**	$K^+[C_{10}H_8]^-$	HOTf	0.95	75.4	80.1	0.65	5.0 (3)	5.0
			1.1	84.7		0.68	5.2 (3)	
			1.0	80.1		0.61	4.7 (3)	

[a] All reactions were carried out in THF under N_2 at -78 °C. [b] [complex] = 6.7 × 10^{-3} M. [c] Quantification of NH_4^+ was calculated by ^{1}H NMR method. [d] [2,5-dimethyltetrahydrofuran (std)] = 5.2 × 10^{-2} M. The integration values of NH_4^+ are shown against the vinyl protons of 2,5-dimethylfuran (2H). [e] Yields are based on a chromium ion. [f] Quantifications of N_2H_4 were calculated by *p*-dimethylaminobenzaldehyde method. [g] This value was obtained from absorbance of the peak at 458 nm of a yellow azine dye. [h] Yields are based on a vanadium ion.

(1)

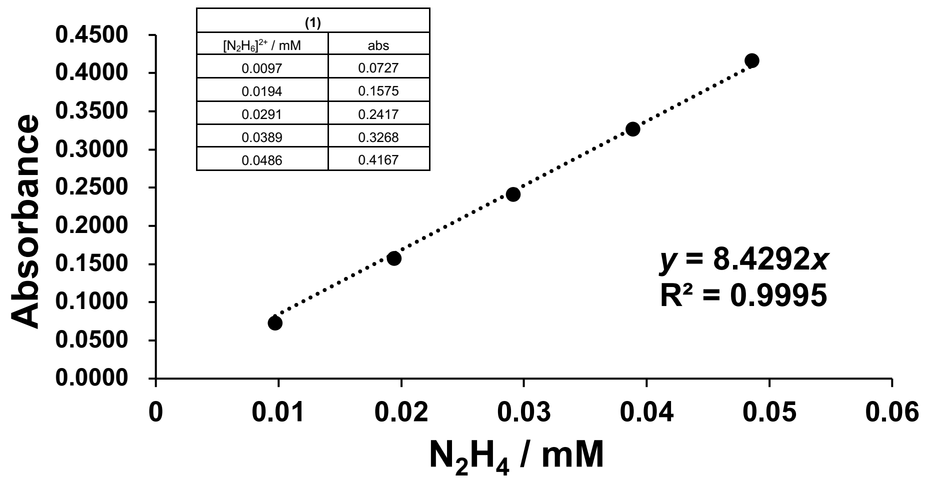

(2)

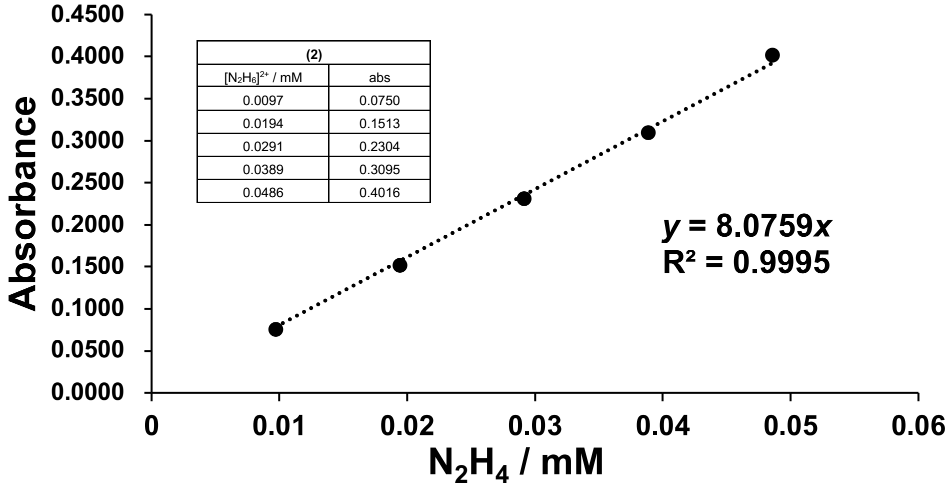

(3)

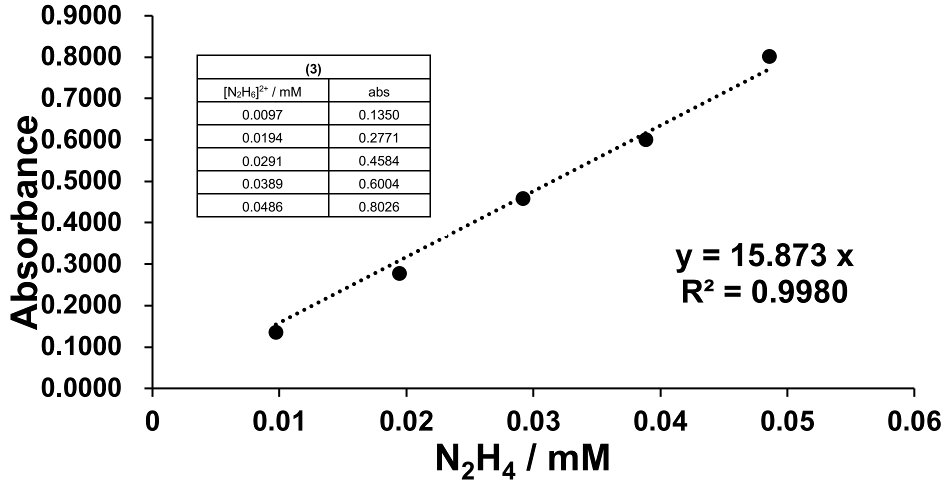

Figure S16. Calibration curves for hydrazine quantification.
